# Supplementary material for: Splice-Junction-Based Mapping of Alternative Isoforms in the Human Proteome
Source: Cell Rep. Author manuscript; Available in PMC 2020 Jan 15. (PMC6961840; doi:10.1016/j.celrep.2019.11.026)
Supplement: 2 [file NIHMS1546469-supplement-2.zip › DF1/PXD006675/LeftAtrium/LeftAtrium_48_PLEKHH3_GALEPGPGQEAGGAGPAR.pdf]

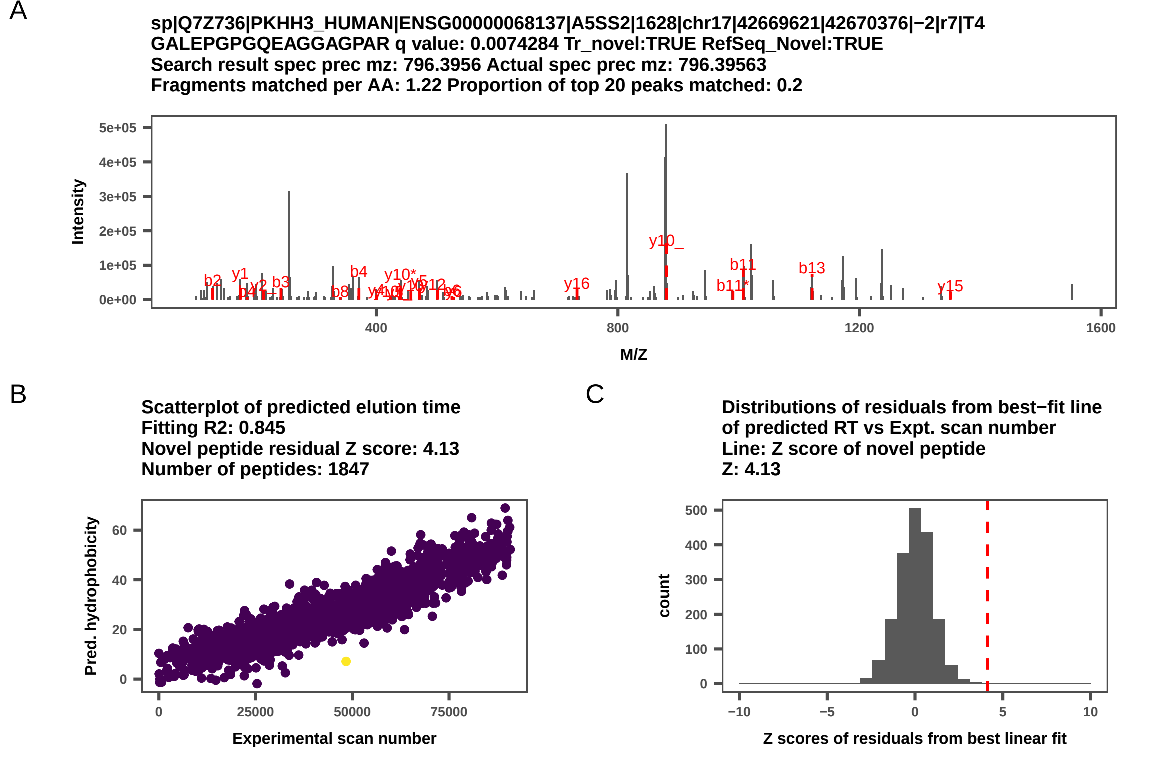

A
sp|Q7Z736|PKHH3_HUMAN|ENSG00000068137|A5SS2|1628|chr17|42669621|42670376|−2|r7|T4
GALEPGPGQEAGGAGPAR q value: 0.0074284 Tr_novel:TRUE RefSeq_Novel:TRUE
Search result spec prec mz: 796.3956 Actual spec prec mz: 796.39563
Fragments matched per AA: 1.22 Proportion of top 20 peaks matched: 0.2
Intensity
5e+05
4e+05
3e+05
2e+05
1e+05
0e+00
b2
y1
y2
b3
b4
y10*
y5
b8
y16
y10_
b11
b11*
b13
y15
400
800
1200
1600
M/Z
B
Scatterplot of predicted elution time
Fitting R2: 0.845
Novel peptide residual Z score: 4.13
Number of peptides: 1847
Pred. hydrophobicity
60
40
20
0
0
25000
50000
75000
Experimental scan number
C
Distributions of residuals from best−fit line
of predicted RT vs Expt. scan number
Line: Z score of novel peptide
Z: 4.13
count
500
400
300
200
100
0
−10
−5
0
5
10
Z scores of residuals from best linear fit
